# Subjective Time in Dementia: A Critical Review

**DOI:** 10.3390/brainsci11111502

**Published:** 2021-11-12

**Authors:** Lulu Liu, Adam Bulley, Muireann Irish

**Affiliations:** 1 School of Psychology, The University of Sydney, Sydney, NSW 2006, Australia; lulu.liu1@sydney.edu.au (L.L.); adam.bulley@sydney.edu.au (A.B.); 2 Brain and Mind Centre, The University of Sydney, Sydney, NSW 2050, Australia; 3 Department of Psychology, Harvard University, Boston, MA 02138, USA

**Keywords:** neurodegenerative disorders, Alzheimer’s disease, frontotemporal dementia, time perception, mental time travel, prospective memory, delay discounting, intertemporal decision-making, episodic memory

## Abstract

The capacity for subjective time in humans encompasses the perception of time’s unfolding from moment to moment, as well as the ability to traverse larger temporal expanses of past- and future-oriented thought via mental time travel. Disruption in time perception can result in maladaptive outcomes—from the innocuous lapse in timing that leads to a burnt piece of toast, to the grievous miscalculation that produces a traffic accident—while disruption to mental time travel can impact core functions from planning appointments to making long-term decisions. Mounting evidence suggests that disturbances to both time perception and mental time travel are prominent in dementia syndromes. Given that such disruptions can have severe consequences for independent functioning in everyday life, here we aim to provide a comprehensive exposition of subjective timing dysfunction in dementia, with a view to informing the management of such disturbances. We consider the neurocognitive mechanisms underpinning changes to both time perception and mental time travel across different dementia disorders. Moreover, we explicate the functional implications of altered subjective timing by reference to two key and representative adaptive capacities: prospective memory and intertemporal decision-making. Overall, our review sheds light on the transdiagnostic implications of subjective timing disturbances in dementia and highlights the high variability in performance across clinical syndromes and functional domains.

Despite being an intimate and fundamental feature of human psychology, the subjective experience of time remains poorly understood. Whether crossing the road, watching the clock during a meeting, anticipating when to take medication, or planning an upcoming appointment, everyday adaptive functioning relies on dependable cognitive mechanisms for subjective timing. These mechanisms enable people not only to fine-tune everyday goal-directed behaviour, but also to anticipate the best course of action over longer timescales; to adjust, predict, and refine behaviours with the long-term future in mind [1,2,3,4,5]. In turn, the subjective perception of time, along with the ability to navigate mentally through time (re-experiencing past events and imagining possible future occurrences), gives rise to a sense of self-continuity across past, present, and future contexts [6,7].

Subjective time is not a unitary construct. Instead, it draws upon a number of interacting capacities including attention, working memory, and episodic memory [3,8,9]. Given this complexity, lapses in subjective timekeeping are commonplace, and reflect the breakdown of different underlying components. This is observable across diverse neuropsychiatric and clinical conditions ranging from schizophrenia [10,11] to post-traumatic stress disorder [12]. In recent decades, mounting evidence has suggested that neurodegenerative disorders present with significant disruptions to both time perception and mental time travel (see Glossary, Box 1). Given that these capacities are linked to adaptive everyday functions—from driving to financial saving—understanding their vulnerability in neurodegenerative disorders is an essential goal for contemporary psychology, neuroscience, and neurology. The objectives of the current narrative review are to explore how subjective time is compromised in dementia syndromes—focusing on Alzheimer’s disease (AD) and frontotemporal dementia (FTD)—and to elucidate the neurocognitive mechanisms potentially driving such disturbances. We further consider the functional implications of altered subjective timing by reference to two representative adaptive capacities—prospective memory and intertemporal decision-making—along with key suggestions for future research.

Box 1. **Glossary.**

**Cognitive offloading:** the use of physical action or external tools to alter the information processing requirements of a task and reduce internal cognitive demand.**Metacognition:** cognition about cognition; the capacity to monitor, evaluate, and control one’s own cognitive processes.**Core network:** a network of brain regions that show increased activity both when people remember past experiences and when they imagine future experiences.**Prospective memory:** the ability to remember to carry out intentions in the future.**Delay discounting:** the decline in the subjective value of an outcome with the delay to its receipt.**Retrospective time judgment:** subjective timing judgments made at the end of an experiment without prior instructions to keep track of time.**Duration discrimination:** deciding whether a comparison duration is shorter or longer than a presented duration.**Time estimation:** tasks requiring participants to estimate how long stimuli were presented.**Episodic future thinking:** the capacity to imagine or simulate experiences that might occur in one’s personal future.**Time perception:** the ability to perceive, judge, and represent time intervals.**Episodic memory:** recollection of personally experienced events situated within a unique spatial and temporal context.**Time production:** the requirement to produce or generate experimenter-specified durations.**Intertemporal decision-making:** making decisions with consequences that play out only over time, often involving trade-offs between sooner and later costs and benefits.**Time reproduction:** the ability to reproduce specific durations presented by an experimenter.**Mental time travel:** the capacity to mentally navigate through subjective time, including episodic memory and episodic foresight/future thinking.

## 1. Time Perception

### 1.1. The Foundations of Time Perception

Broadly defined, time perception refers to the capacity to perceive, judge, and represent time intervals [9,11,13]—a ubiquitous occurrence in daily life. Consider the myriad timing processes involved in cooking a meal—one may need to wait several seconds for butter to melt, judge when several minutes have passed to remove a pot from the stove, or estimate the total time required to finish the recipe. While external aids such as timers or alarms can be used to support timing processes as a form of cognitive offloading [14], the tracking of relatively short durations nevertheless draws upon core cognitive processes, including attention and memory [15].

**Figure 1:**
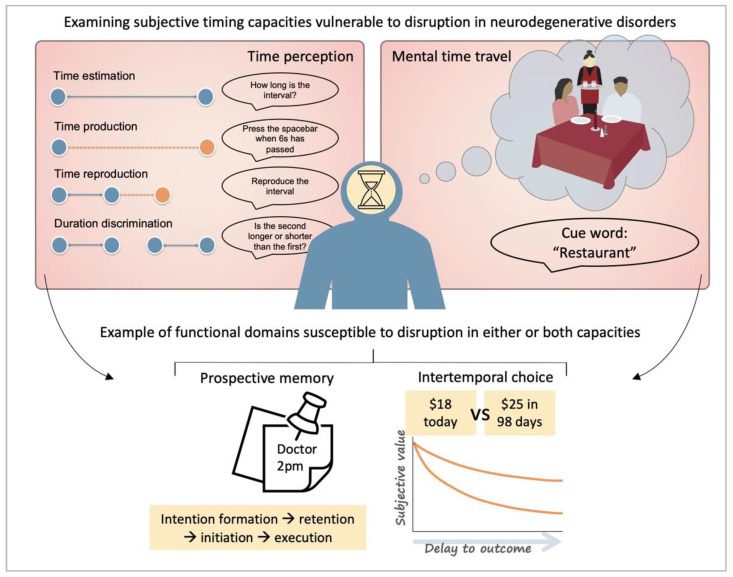
Mechanisms of subjective time disruption in dementia span time perception and mental time travel capacities. Many essential functional domains rely on both capacities, such as prospective memory (remembering to perform an intention) and intertemporal choice (making decisions with outcomes that play out over time). Public domain restaurant image from Openclipart.

In the laboratory, time perception is typically studied using experimental tasks spanning short-term durations in the order of milliseconds, seconds, or minutes (reviewed by [9]). These assessments include time estimation (estimating how a long stimulus was presented for), time production (producing a specified duration), time reproduction (reproducing the same duration as a presented stimulus), and duration discrimination (deciding whether a comparison duration is shorter or longer than a standard duration) (Figure 1). Additionally, time perception studies explore temporal relations between events [16], such as temporal simultaneity (i.e., whether two events are in sync [17]), and temporal order (i.e., which of two events occurred first or second [18]). Collectively, the capacities assessed by these tasks enable people to accommodate changing representations of temporal context; to form and update a dynamic structure of the external environment [19].

### 1.2. Neural Substrates of Time Perception

**Figure 2:**
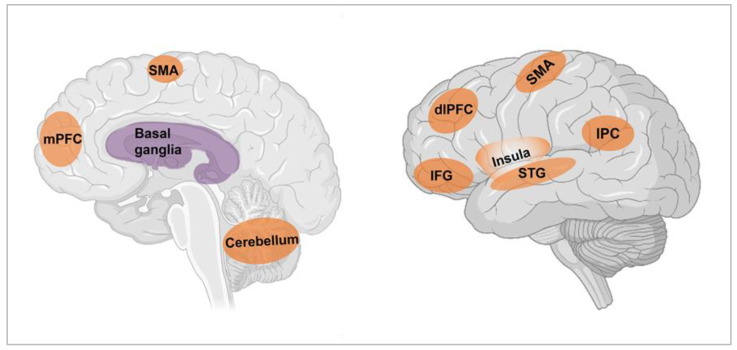
Brain regions typically implicated in prospective time perception, as revealed by fMRI studies in healthy young adults. dlPFC: dorsolateral prefrontal cortex; IFG: inferior frontal gyrus; IPC: inferior parietal cortex; mPFC: medial prefrontal cortex; SMA: supplementary motor area; STG: superior temporal gyrus. Brain template by BioRender.com (accessed on 22 March 2021).

On the neuroanatomical level, fMRI studies of time perception in healthy participants point to the coordinated activity of multiple brain regions within a distributed cortico-subcortical brain network [20,21,22,23], although there is some debate as to the modality-specific versus domain-general nature of this activation [24]. Here, we summarise the main regions consistently engaged across studies of subjective timing (Figure 2); for a recent review, see [20]. The main frontal regions implicated include the superior, medial, and inferior frontal gyri (including the dorsolateral prefrontal cortex and ventrolateral prefrontal cortex) [25], along with the precentral gyrus and anterior cingulate gyrus. These regions play an established role in cognitive control and sustained attention processes, necessary for successful completion of timing tasks [26]. Activation in the supplementary motor area on timing tasks has been associated with cognitive functions such as attentional allocation, with the suggestion of a rostrocaudal gradient that might be sensitive to timing duration [27]. Meanwhile, the observation of insula activation, across timescales and timing tasks (i.e., motor, perceptual), has been interpreted in favour of the direct encoding of time intervals by the posterior insula [28], while anterior insular involvement has been linked to salience detection and the integration of emotional and arousing representations to construct a subjective awareness of time [29]. Regions such as the superior temporal gyrus and the cerebellum have been implicated in modality-specific aspects of timing, such as auditory [30] and motor preparation and prediction, respectively. Activation of inferior parietal regions such as the precuneus and supramarginal gyrus may further reflect the role of attentional processes during timing [31]. Finally, subcortical structures in the basal ganglia—including the caudate and putamen—are reliably engaged during time-perception tasks [32,33]. The caudate is known to be active in timing processes for second-range intervals [9], either detecting deviant durations in patterns of stimuli [23] or discriminating between two temporal stimuli [34]. In contrast, the putamen has been shown to support the production of remembered durations, which might relate to working memory or the precise timing of a motor plan [23]. The perception of time is evidently supported by coordinated activity within a widely distributed cortico-subcortical network, key nodes of which are vulnerable to injury or degeneration [35,36].

## 2. Mental Time Travel

### 2.1. The Foundations of Mental Time Travel

Whereas time perception typically refers to the apprehension of short durations (i.e., sub-seconds, seconds), mental time travel refers to the ability to navigate mentally through extended periods of subjective time [4,37]. This capacity enables one to revisit events from the past, via autobiographical or episodic memory, or to project oneself into the future via episodic foresight or future thinking [4,38,39]. These temporally extended voyages across past and future contexts rely upon episodic and semantic representations that can be flexibly recombined into novel configurations [3,40,41]. A defining feature of mental time travel is the temporal information associated with the event representation, including information about temporal distance, context, or order [42,43,44]. Temporal distance refers to the length of time between the specific event and the present (e.g., “Christmas happened three months ago”), while temporal context anchors events in the past, present, or future. Temporal order refers to relations between events (e.g., one event happened before another event), such as remembering that one became ill following a particular meal rather than the other way around. Mental time travel enables us to navigate seamlessly back and forth through subjective time, to reorient ourselves to the present moment, and to be aware of current temporal dynamics. As will become apparent, mental time travel also underwrites a range of important adaptive functions, including emotion regulation and spatial navigation [3], anticipating and preparing for future threats [45,46,47], flexible decision-making [48], as well as maintaining a continuity of selfhood across one’s subjective timeline [6,7].

### 2.2. Neural Substrates of Mental Time Travel

In terms of neural architecture, the capacity for mental time travel relies on the integrity of a core network that overlaps closely with the default mode network of the brain [49]. Anchored on the medial temporal lobes—notably, the hippocampus—this core network plays a pivotal role in the reconstruction of past events and the construction of future scenarios [50,51]. Hippocampal contributions to past and future thinking have primarily been interpreted in relation to the extraction of episodic details from past experiences and their flexible recombination into novel future events [52]. The hippocampus is also heavily implicated in the construction of spatially coherent scenes or layouts, which provide the spatial backdrop for past and future event constructions [50], yet has further been suggested to represent temporal order information and to support the temporal organization of memories [53,54]. However, the hippocampus does not work in isolation, and benefits from close functional coupling with the ventromedial prefrontal cortex (vmPFC) [55]. While its precise role in mental time travel remains unclear, vmPFC engagement has been proposed to reflect the instantiation of appropriate schemas or knowledge structures [56], while other studies suggest that the vmPFC supports the integration of social or affective content into an event simulation [57,58]. More recently, it has been suggested that the vmPFC initiates the construction of mental scenes by coordinating the assimilation of perceptual details from neocortical sites [55]; however, activation of anterior midline regions might also reflect self-referential processing across subjective time [59].

Rostrolateral prefrontal activation likely reflects the ongoing cognitive control processes required to coordinate the online maintenance of temporally extended past and future representations [60]. Interestingly, considerable posterior parietal cortical involvement has been observed during past and future simulation [61,62,63], reflecting the importance of a posterior parietal memory network comprising the posterior parahippocampus, retrosplenial cortex, and posterior cingulate cortex [64], implicated in contextual association processing [65] and coding for the presence of space [66]. Lateral parietal activation—particularly in the angular gyrus—has recently been suggested to reflect the multimodal integration of sensory–perceptual details into a contextually rich layer [67], which is then overlaid onto the core memory supplied by the hippocampus [68]. Finally, lateral temporal lobe involvement likely provides the requisite semantic knowledge to scaffold the constructed event, into which episodic details can be assimilated [40,69]. From this brief summary, the complexity of the neurocognitive architecture of mental time travel should be evident. We consider next how this complexity renders subjective timing particularly vulnerable in the context of neurodegenerative disorders.

## 3. Why Study Subjective Time in Neurodegenerative Disorders?

**Figure 3:**
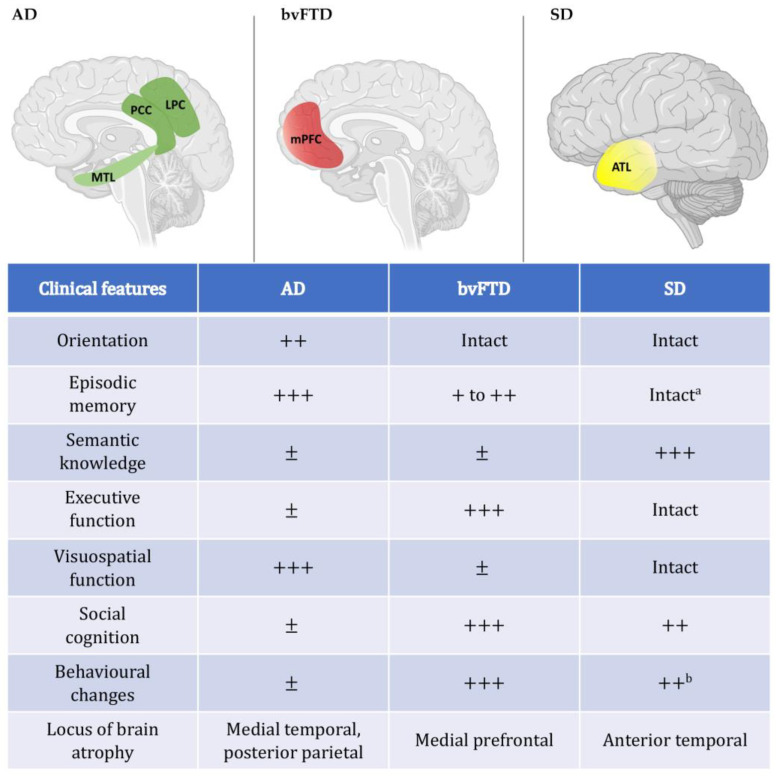
Top panel displays canonical epicentres of atrophy in the clinical syndromes of interest. AD: Alzheimer’s disease; bvFTD: behavioural-variant frontotemporal dementia; SD: semantic dementia; ATL: anterior temporal lobe; mPFC: medial prefrontal cortex; MTL: medial temporal lobe; LPC: lateral parietal cortex; PCC: posterior cingulate cortex. Brain template accessed on 22 March 2021, from BioRender.com. Table provides an overview of characteristic clinical profiles in each dementia syndrome. +: mild impairments; ++: moderate impairments; +++: severe impairments; ±: variable performance depending on method of assessment; Intact: not significantly different to healthy older control performance. ^a^ Function is intact when non-conceptually loaded test materials are used. ^b^ Emerges with disease progression.

Neurodegenerative disorders are characterised by progressive impairments in cognition, behaviour, and/or motor function due to the degeneration of large-scale functional brain networks [35,70]. This variable deterioration of distributed brain networks gives rise to a constellation of higher order cognitive and behavioural changes, many of which bear relevance to the domains of time perception and mental time travel [36,71]. To date, studies exploring alterations in subjective time in neurodegenerative disorders have tended to focus on Parkinson’s disease [72,73,74]. This is perhaps unsurprising when we consider that the locus of pathology in Parkinson’s disease resides in the basal ganglia—one of the main subcortical regions implicated in subjective timing in healthy adults [75]. However, much is still not understood about time perception and mental time travel in neurodegenerative disorders more broadly, especially in the context of dementia syndromes. Here, we consider the conditions of Alzheimer’s disease (AD), characterised by marked episodic memory dysfunction; behavioural-variant frontotemporal dementia (bvFTD), in which executive dysfunction and behavioural changes predominate; and semantic dementia (SD), the hallmark feature of which is an amodal semantic impairment (see Figure 3). We will next argue that subjective timing disturbances manifest variably across these clinical syndromes, and may account for a range of behavioural and functional impairments exhibited by patients in their daily lives.

### Clinical Reports of Subjective Timing Difficulties in Dementia

Clinical and anecdotal evidence indicates distinct changes in subjective timing in Alzheimer’s disease (AD), in parallel with canonical episodic memory difficulties [71]. Patients typically present to the clinic displaying disorientation to time and place, manifesting in a loss of awareness about the current date and time period [76]. While the fundamental semantic knowledge of time appears relatively preserved in AD (e.g., how many minutes are in an hour) [77], difficulties reading and comprehending time emerge with disease progression [78]. Disruption to prospective and retrospective time perception has been suggested to deleteriously impact the capacity for past and future mental time travel in AD [79]—a topic to which we return in more detail later.

Although time perception represents a nascent topic in frontotemporal dementia, we argue that there is sufficient clinical and anecdotal evidence to warrant consideration of subjective timing disturbances in these syndromes. Patients with the behavioural variant of frontotemporal dementia (bvFTD)—a younger onset neurodegenerative disorder characterised by personality and behavioural changes—generally remain oriented to time and place [80]. Interestingly, however, patients with bvFTD display increased temporal rigidity in their everyday behaviours—for example, insisting on performing household tasks in exactly the same order each time [81]. More striking is the recent finding of extreme environmental dependency in bvFTD, whereby patients appear unable to perceptually decouple from the current moment in order to engage in endogenously driven past or future forms of spontaneous cognition [82]. This inability to revisit events from the past or to envisage the future results in the individual becoming increasingly tethered to the present moment in time, and may underlie a range of behavioural disturbances that typify this syndrome [7,83].

Finally, semantic dementia (SD) provides a rare opportunity to explore how the progressive deterioration of the conceptual knowledge base impacts the fundamental understanding of the construct of time and the capacity to mentally navigate across past and future temporal contexts [84]. Clinical observations indicate that while SD patients remain well oriented to time and place [85], patients become increasingly bound by routines—for example, insisting on doing things at a particular time, or displaying discomfort when time schedules are disturbed [81]. A proclivity for clock watching is also observed [86], with recent evidence suggesting a preoccupation with time in some SD cases [87].

Elucidating the precise clinical manifestations of these disturbances represents an important pursuit, enabling us to shed light not only on the fundamental mechanisms of subjective timing, but to also pave the way towards the development of practical guidelines to manage the everyday consequences of these symptoms.

## 4. Time Perception in Dementia

### 4.1. Prospective Timing in Alzheimer’s Disease

Experimental investigations of time perception in dementia typically employ measures of prospective timing, including time estimation, time production, time reproduction, and duration discrimination (see Box 1). Across such tasks, AD patients commonly show impaired accuracy, higher error rates, and increased performance variability, with the suggestion that such impairments are more pronounced in estimation and reproduction tasks [88,89,90]. In an early study, Nichelli et al. explored the accuracy and precision of time perception in a sample of mild-to-moderate AD patients. Using a verbal estimation procedure, participants were asked to read 5, 10, 20, or 40 digits, one at a time, while concurrently reproducing a standard interval of one key press per second [89]. Following each sequence, participants then estimated the length of time elapsed from the start of the trial. Relative to healthy older controls, AD patients showed poorer accuracy and precision in the time estimation task, with variable performance on the time reproduction condition. This profile of increased variability and decreased accuracy in AD has since been replicated using various time perception measures. For example, Carrasco et al. employed a time production task whereby participants were required to produce three distinct time intervals (5 s, 10 s, and 25 s) using the space bar to denote the beginning and end of each estimated interval. Relative to healthy older adults, patients with AD displayed reduced accuracy across all time intervals [91]. Similarly, a later study using a verbal time estimation task reported more absolute errors and greater performance variability in AD patients relative to older adults [90]. More recently, El Haj et al. [79] instructed patients with AD to read a series of numbers for varying durations of time (30 s, 60 s, 90 s, and 120 s) and then to estimate the duration spent reading. Irrespective of duration, AD patients were found to underestimate time durations compared to both younger and older adults.

The origins of variable performance on timing tasks in AD have been further explored using manipulations of time duration and task complexity. Using time bisection tasks, Caselli et al. [92] required mild AD patients to adjudicate on whether different time durations (sub-second range: 100–600 ms; supra-second range: 1000–3000 ms) were shorter or longer relative to a reference interval. While longer time bisection capacity was not found to differ between AD and younger or older control groups, AD patients displayed increased variability in the timing of millisecond durations [92]. Papagno et al. instructed participants to give verbal estimations of time durations (15 s, 50 s) while completing various concurrent tasks of attention and short-term memory [93]. AD patients consistently overestimated time intervals, irrespective of duration, relative to age-matched healthy controls, which in turn was attributed to the role of executive and attentional factors [93]. El Haj et al. considered the role of task complexity by requiring participants to perform time reproduction tasks during high and low attention-demanding conditions [88]. Overall, timing performance decreased as task complexity increased, whereby AD patients under-reproduced the time duration on the high- relative to the low-attentional conditions.

The evidence to date suggests a profile of increased variability and decreased accuracy, in the context of marked heterogeneity in AD on prospective time-perception tasks, with some patients overestimating and others underestimating time intervals. How such profiles relate to disease staging and level of cognitive impairment remains unclear; however, it is likely that episodic memory and attentional difficulties play prominent roles [71].

### 4.2. Retrospective Timing in Alzheimer’s Disease

While the bulk of experimental studies have focused on prospective timing capacities in AD, there is some evidence to suggest that retrospective judgments may also be compromised. Retrospective time judgments require participants to provide an estimate of the time elapsed (usually in tens of minutes), typically at the conclusion of an experiment and without any prior warning to keep track of time. An interesting study in this regard was conducted by Heinik, who explored the capacity for retrospective timing in 16 AD and 11 vascular dementia patients [94]. Participants completed a series of multiple cognitive assessments, which lasted for a total duration of 67 minutes, and were subsequently asked to retrospectively estimate the duration of the test session. Interestingly, patients with dementia were not found to differ from healthy older participants, suggesting that retrospective time judgements may be relatively preserved, at least in the early stages of the AD disease course [94].

From these studies, we can surmise that prospective and retrospective timing capacities are not uniformly impaired in AD—a proposal that is borne out in a recent meta-analysis of timing disturbances in mild cognitive impairment (MCI) and AD [95]. Although multiple findings suggest aberrant time perception in AD, a direct comparison across different methodologies is lacking, as is a systematic examination of how subtle changes in task design impact timing performance in dementia [9]. In terms of underlying mechanisms, while some studies have proposed a role for an internal clock change, working memory, or attentional factors [71,92,93], formal testing of these mechanisms has failed to establish reliable contributors [90]. In the same vein, the neural substrates of disrupted timing in AD remain to be established. Future work pairing structural and functional neuroimaging approaches will be necessary to delineate the neurocircuitry of time perception impairments across the disease trajectory in AD.

### 4.3. Prospective Timing in Frontotemporal Dementia

While a large body of evidence indicates prominent alterations in time perception in AD, studies in frontotemporal dementia (FTD) are comparatively sparse. This gap in knowledge is somewhat surprising given reports of temporal rigidity and adherence to routines in these syndromes [86,87]. In a single case study, Wiener and Coslett explored time perception performance in a patient with probable FTD—most likely the behavioural variant of FTD (bvFTD)—to test the claim that the frontal lobes are crucial for subjective timing [96]. A battery of timing tasks was administered covering duration estimation, time production, and reproduction abilities across supra-second intervals (2–12 s), temporal discrimination of sub- (300–600 ms) and supra-second (2–8 s) intervals, along with a finger-tapping task, while line length estimation, production, and reproduction tasks served as non-temporal control tasks. Relative to an age-matched control group, the FTD patient displayed poorer accuracy on time estimation and production tasks, resulting in under-production and rather extreme over-estimation of time intervals. Normal accuracy was observed for time reproduction in the context of heightened performance variability, while duration discrimination and finger-tapping performance did not differ from control participants. Importantly, the FTD patient displayed preserved judgment on the non-temporal tasks, suggesting a specific impairment in discrete aspects of prospective timing. The authors proposed that such alterations in timing might reflect a faster internal clock mechanism, while working memory difficulties might further impede task performance, although these proposals were not formally tested.

Building on the initial clues provided by the case study by Wiener and Coslett, a more recent group study provided further definitive evidence of timing disruption in bvFTD, as well as possible underlying mechanisms [97]. Henley et al. administered a battery of timing tasks to a group of dementia patients comprising 20 bvFTD, 11 semantic dementia (SD), 4 progressive non-fluent aphasia (PNFA), and 8 AD patients [97]. Motor timing was measured using a finger-tapping task under externally paced and self-paced conditions. During the externally paced condition, participants were required to make 50 key taps on beat with audio tones presented at a fixed interval of one tone every 1500 ms. By contrast, the self-paced condition more closely reflected a time reproduction task, whereby a short succession of tones was presented at a fixed interval of 1500 ms and then ceased, following which participants were required to tap the key, self-paced, for 50 additional key taps to the same beat. Relative to a control group, bvFTD patients displayed heightened variability in both the self-paced and externally paced tapping conditions, and were more likely to “drift” steadily away from the proper pace, becoming progressively faster or slower. Notably, self-paced performance decrements were associated with working memory capacity, while externally paced performance impairments were found to correlate with executive function. Collectively, these results offer important insights into the mechanisms by which bvFTD patients might experience time disruption.

Despite evidence for an increased interest in time and a propensity for clockwatching, little research has explored prospective or retrospective timing in SD. To our knowledge, the report by Henley et al. [97] remains the only study in which time perception has been formally assessed in this syndrome. Interestingly, no significant differences were exhibited by SD patients relative to controls across any of the measures of time perception, in either the self-paced or externally paced conditions [97]. We tentatively interpret these findings as supportive of a general preservation of timing in SD, at least over short temporal durations. This proposal is corroborated by the intriguing observation of relatively intact time-based prospective memory in SD [98]—a finding we will consider in more detail below. The topic of time perception in SD currently remains ripe for exploration, particularly with a view to identifying the mechanisms that support this relatively intact capacity.

## 5. Disrupted Capacity for Mental Time Travel in Dementia

### 5.1. Revisiting the Past

The capacity to mentally travel back in subjective time to revisit defining events from one’s past is often considered a prototypical expression of the self, and one that is particularly vulnerable to disruption in dementia [7,99]. Autobiographical memory (ABM) enables us to recollect personally relevant memories situated at unique moments in time that are replete with sensory–perceptual details and emotional connotations, conferring a sense of autonoetic (self-knowing) reliving [100,101]. Disruption to ABM in dementia not only impacts an individual’s sense of self, but has been shown to also impact negatively on the patient–carer relationship [102].

Early studies of ABM dysfunction in AD demonstrated a temporally graded pattern of retrieval in accordance with Ribot’s Law [103], with impoverished recent recall in the context of relatively spared remote memory [104,105,106,107,108]. However, with the advent of assessment tools using uncapped scoring methods [109], reports of flat profiles of retrieval in AD emerged, with recent and remote memories comparably affected [110,111,112,113]. Interestingly, discrete islands of preservation persist in the face of the AD pathological process—most notably older, more temporally distant memories that are likely to be semanticised and to carry self-defining personal semantic information [7]. Importantly, the phenomenological experience of mental time travel to the past is also compromised in AD, with patients reporting distinct alterations in the vividness, self-referential quality, and emotional re-experiencing of formerly evocative events [107,114]. Patients gradually lose the capacity to mentally relive their past experiences, but instead report simply “knowing” that these events have taken place [107,108,115]. Closer inspection of the ABM narrative in AD further reveals a definitive shift in tense production, with patients defaulting to a present- rather than past-oriented narrative style [116]. As such, not only is the capacity for mental time travel to the past compromised in AD, but so too is the mode by which AD participants engage in and narrate their past events.

There is now sufficient evidence to conclude that mental time travel to the past is also grossly compromised in bvFTD. While episodic memory impairments are not typically considered a core diagnostic feature of bvFTD [117], studies of ABM consistently reveal marked alterations in this capacity. Importantly, these ABM impairments in bvFTD span all time periods, culminating in a flat retrieval profile [108,111,112,113,118,119,120]. The recollective experience is also compromised, with bvFTD patients demonstrating significantly reduced autonoetic reliving of the past [108]. This global impairment in retrieving specific events from the past is further compounded by an inability to generate rich contextual details during event elaboration. Notably, this paucity of detail encompasses spatial and temporal information for recent events, such that while patients remain well oriented to time and place in general, they seem unable to access the relevant spatiotemporal details from memory when describing past experiences [112].

Finally, patients with SD show an intriguing profile of loss and sparing for ABM retrieval, departing dramatically from the negative temporal gradient of AD and the flat gradient observed in bvFTD. Early studies of ABM retrieval in SD indicated a reverse temporal gradient or, more accurately, a step function, whereby recent episodic experiences remain remarkably preserved relative to events from the distant past [108,121,122,123]. This finding has been replicated more recently using ecologically valid tasks that seek to preserve the essential qualities of episodic memory (i.e., the what, where, and when), [124] as well as more elaborate assessments of contextually rich ABM retrieval [112,125]. The consistent finding to emerge across these studies is of a relative preservation of recent experiences, in contrast with a marked impairment in the retrieval of remote memories [40,126]. This detailed recollection of recent experiences is accompanied by a preserved sense of mental reliving [108], suggesting a preferential encoding and re-experiencing of past events provided they are temporally close to the present day. The temporal boundaries that distinguish recent from remote experiences remain a source of debate [127], and the process by which older memories become semanticised over time remains unclear. What is becoming apparent, however, is that the truncation of the temporal window to that of recently experienced events in SD has important functional implications. Strikwerda-Brown et al. propose that many of the seemingly inflexible and stereotypical behavioural changes observed in SD might reflect the anchoring of the self to recently experienced events [7]. As the self becomes progressively more situated within the present tense [116], patients with SD might increasingly rely on recent experiences to guide their behaviour, manifesting in a strong preference for routine.

### 5.2. Imagining the Future

**Figure 4:**
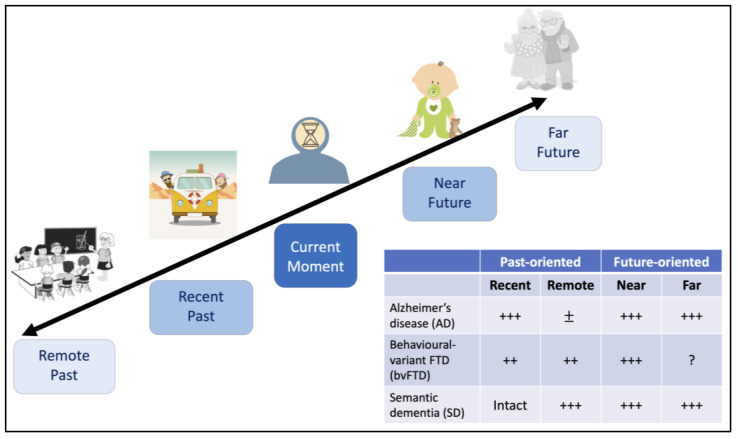
Bending time’s arrow—mental time travel allows us to revisit past experiences and to envisage possible future events, and is markedly compromised in dementia. Schematic showing how temporal distance from the current moment influences past retrieval and future thinking in healthy young adults. Images sourced from Pixabay.com (accessed on 22 March 2021). The accompanying table summarises the existing literature on mental time travel disturbances in dementia. *Notes.* ++: moderate impairments; +++: severe impairments; ±: variable performance depending on method of assessment; Intact: not significantly different to healthy older control performance; ?: no empirical data available to date.

While a large corpus of research demonstrates significant impairments in mental time travel to the past in dementia, our mental timelines also extend to the future, and this is likewise vulnerable to impairment (Figure 4). From an evolutionary perspective, the broad capacity to anticipate events that might occur in the future—so-called “prospection”—confers immense adaptive value and flexibility across a diverse range of functions [1,128,129,130,131]. Although a relatively newborn field, there is now sufficient evidence to conclude that the ability to mentally travel forwards in subjective time to envisage the future is deleteriously affected in dementia [84].

Studies of future thinking in AD demonstrate a parallel impairment in the construction of contextually rich events across past and future contexts [132,133,134,135]. These symmetries across temporal conditions have been interpreted as reflecting a compromised capacity to extract sensory–perceptual details from past experiences, and to flexibly recombine these details into novel future scenarios [132]. Interestingly, the phenomenological experience is also affected, with patients rating their past and future events comparably in terms of emotional intensity and personal significance [132,136]. As the majority of studies to date have focused on the simulation of future events occurring 1 year into the future, it remains unclear whether increasing temporal distance from the present disproportionately impacts future thinking in AD. Studies exploring the capacity for semantic prospection (i.e., envisaging non-personal scenarios occurring 10 years into the future) hint at the possibility of a global disruption to any form of future simulation, irrespective of content and temporal distance [84,137].

Comparatively less is known regarding the ability to envisage future events in bvFTD, as to the best of our knowledge only two studies have explored this topic to date. Irish et al. investigated past and future thinking in bvFTD and revealed symmetrical deficits in the construction of episodic events across both temporal contexts, mirroring performance in an AD group [135]. Interestingly, future thinking impairments in bvFTD were found to relate to frontopolar brain atrophy, converging with a large body of work implicating the frontal poles in prospective memory, goal-directed behaviour, counterfactual thinking, and planning [138]. In a subsequent study, the capacity to envisage non-personal information in the distant future was probed, again revealing parallel deficits across past and future contexts in bvFTD [137]. Collectively, these findings point to gross disturbances in temporal processing that extend from the past to the future. As we will discuss shortly, the interplay between prospection difficulties and functional outcomes in bvFTD represents an important area for empirical investigation, given that many of the canonical features of this syndrome would seem to indicate a lack of regard for the future consequences of actions [1].

Finally, studies of future thinking converge to reveal an asymmetric impairment of future relative to past forms of thinking in SD [139,140]. Despite a relative preservation of recent episodic retrieval, patients with SD display marked difficulties in envisaging and describing events located in the near future in rich contextual detail [136,141,142]. This future-oriented impairment spans multiple representational formats, including representation of oneself [141], construction of detailed self-referential future scenarios [142], and simulation of non-personal public events that might occur in the far future [142]. These pervasive impairments in future-oriented thinking have been interpreted in relation to the profound pan-modal loss of conceptual knowledge that typifies this syndrome [84,139]. In the absence of the appropriate semantic scaffold, patients with SD lack the conceptual framework upon which to construct their future events [140]. Relatedly, provision of an appropriate scaffold can support the capacity for prospection in SD, as demonstrated by a recent fMRI study in which patients with SD could envisage future events within the next 12 months that had been pre-selected by family members [143]. This finding resonates with reports of relatively intact atemporal forms of imagination in response to commonplace predetermined cues in left-lateralised cases of SD [144], suggesting that the de novo construction of events located in the future may be particularly vulnerable in SD as opposed to imagination writ large.

Whether temporal distance plays a modulating role in this context remains unclear, though a recent case study of a patient with SD sheds some light on this question. Patient SL was asked to remember events in the near (last week), intermediate (1 year ago), and remote (5 years ago) past, and to envisage future events in the corresponding time periods, i.e., near future (next week), intermediate future (1 year’s time), and distant future (5 years’ time). Relative to controls, SL had the greatest difficulty with the most temporally distant past and future conditions, while past and future thinking for near and intermediate temporal contexts remained relatively intact [145]. While group studies will be necessary to confirm this finding, it nevertheless provides important insights regarding the effects of temporal distance on mental time travel capacities in SD and speaks to the increasing role of semantic memory in modulating past and future thinking as we move further away from the present moment in time [40,127].

## 6. Functional Relevance of Subjective Time Disturbances in Dementia

Studies on subjective time in dementia syndromes remain relatively sparse and are constrained by the laboratory setting. Deficits in time perception and mental time travel, however, are predicted to have substantial real-world implications given the ubiquity of timing-related activities in everyday life. Investigating the functional relevance of subjective timing disturbances in dementia is therefore paramount. We explore this functional relevance with reference to two key representative adaptive capacities that are vulnerable to disruptions in time perception, mental time travel, or both: intertemporal decision-making and prospective memory.

### 6.1. Disruptions in Intertemporal Choice

Intertemporal choices are those with consequences that play out over time, involving trade-offs between sooner and later outcomes [146,147]. One well-established phenomenon in intertemporal decision-making is that the subjective value of a delayed outcome tends to become discounted with increasing delays to its occurrence. This tendency for delay discounting is near-universal in adults [148], present in non-human animals [149], and emerges early in childhood [150]. Steeper delay discounting (wherein rewards more quickly lose their subjective value with delays to their receipt) has become a prominent target for applied research, not least because of its occurrence in a range of psychopathologies [151] and associations with outcomes ranging from financial debt [152] to life expectancy [153,154]. Various lines of evidence have implicated subjective timing in the steepness of delay discounting [155,156], with some prominent models placing the subjective perception of time centre-stage as both a key neurocognitive mechanism and an essential individual-differences variable [156,157]. According to such models, an individual who relatively overestimates the duration of delays would perceive future rewards as being relatively more distant, and they should therefore perceive the cost of waiting to be higher. Numerous studies indeed find that individuals who perceive the duration of delays to be longer also tend to more steeply discount delayed rewards [157,158,159], consistent with emerging views of delay discounting as the product of subjective, rather than objective, time delays [157,159,160,161,162].

As we saw earlier in the context of increased timing variability, there is evidence for duration overestimation in cases of both AD and bvFTD (where in at least one study the overestimation was extreme) [96]. For these patients, delays to future rewards may be perceived as being relatively more substantial, making waiting for future rewards less appealing. In line with this notion, a body of empirical research suggests distinct shifts towards steeper delay discounting in neurodegenerative conditions. Across AD and the prodromal stage of MCI, there is evidence for a modest elevation in delay discounting, leading to a greater prioritization of immediate rewards relative to delayed ones e.g., [163,164,165,166,167], although mixed findings exist in the literature (e.g., [168,169,170,171,172,173]). In bvFTD, the evidence is sparser but more consistent, with patients demonstrating substantially steeper delay discounting relative to healthy controls, and in some cases relative to AD [168,174,175] (though see [170,173]). 

In a standard intertemporal choice task, participants make various decisions between smaller amounts of reward available sooner, and larger amounts of reward available later. Usually, these rewards are monetary, although some researchers have also taken steps to develop ecologically valid tasks that involve trading off various goods, such as a choice between a packet of chips now versus a cooked meal in a restaurant in 1 month [169]. When using an ecological intertemporal choice task adapted for dementia, Bertoux et al. [176] found a consistent preference for smaller rewards across every timespan (1 month to 10 years) in patients with bvFTD. Such steeper delay discounting may manifest in the everyday life of bvFTD patients as the consumption of unhealthy but immediately rewarding foods, or a lack of other health-protective behaviours that incur an immediate cost but downstream benefit [176]. These findings resonate with patterns of behaviour common in bvFTD, such as dietary shifts in favour of calorie-dense or high-fat foods [177]. Time perception changes may also account for more subtle shifts in intertemporal decision-making in patient groups. For example, the greater variability in time perception described earlier across dementia syndromes could be associated not necessarily with steeper delay discounting, but with reduced sensitivity to delay information in making intertemporal decisions—a phenomenon observed recently in both AD and bvFTD [168]. Similarly, the noise introduced by variability in subjective time representations could manifest in greater internal uncertainty around estimates of prospective value [178], and therefore less consistency in intertemporal choices (see [179]). Alterations in value-directed learning in bvFTD might further influence responses to intertemporal choice tasks by compromising the capacity to selectively prioritise high-value options [180].

It is an open question how subjective timing mechanisms in the seconds to minutes range relate to those involved over the longer timespans of days or months more common in intertemporal choice tasks. Systems-level accounts of intertemporal decision-making routinely include the core network described earlier, on account of the putative role of mental time travel in managing these longer-term decisions [148,181,182]. In parallel, various models in psychology, economics, and computational neuroscience ascribe a central role to mental time travel as a mechanism for flexibly modifying present choices in line with estimates of the likelihood and value of future outcomes [183,184,185,186,187,188]. Recent research in MCI and AD demonstrates that graded disruptions to both episodic memory and episodic future thinking are associated with steeper delay discounting in these patients [166,167]. In healthy younger adults, promising intervention studies show that cuing participants to imagine future outcomes while making intertemporal choices can reduce delay discounting, both for money as well as for other commodities, such as food, cigarettes, or alcohol [176,189,190,191]. However, older adults [192] and individuals with subjective cognitive decline [193] who exhibit deficits in future imagining show less susceptibility to this episodic future cueing effect. Likewise, individuals with damage to the hippocampus and associated mental time travel deficits through brain injury are not as susceptible to such episodic future cueing effects as healthy controls [194,195], even if they do discount delayed rewards within the normal range [196,197]. The use of personalized, real-life cues may overcome this deficit, allowing individuals with impairments to mental time travel to modify their discounting in response to future event cuing [198,199]. While there is evidence to suggest that neurodegenerative disorders are associated with reduced flexibility in response to related imagery-based cueing protocols [175], it remains to be seen whether alterations to the future event cuing procedure to make it more self-relevant, personalized, and congruent with planned activities will enable reductions to delay discounting in dementia (see [174]).

### 6.2. Prospective Memory Impairments

Prospective memory refers to the process of forming an intention and remembering to carry out that intention in the future [200,201]. Successful prospective memory performance calls upon various cognitive capacities involved in multiple stages, from forming and retaining an intention to subsequently initiating and executing that intention at the right moment [202,203]. Event-based prospective memory concerns the initiation and execution of an intention in response to a particular target event, such as remembering to buy bread when driving past the bakery on the way home from work, while time-based prospective memory concerns the initiation and execution of an intention at a particular time (e.g., leaving the house at 1:30 p.m. for a 2 p.m. appointment) or after a particular amount of time has passed (e.g., remembering to take one’s medicine 30 min after one’s morning meal). Various studies have begun to investigate the contribution of time perception abilities to prospective memory performance, in line with theoretical views that emphasize shared cognitive and neural substrates between these abilities [204]. While some studies implicate time perception accuracy in prospective memory performance [205], other research has failed to find associations between time perception and raw prospective memory performance accuracy per se, instead revealing that time perception abilities relate to the tendency towards time monitoring via checking external aids, such as stopwatches and clocks, as a compensatory strategy to overcome internal deficits to prospective memory [206,207].

Time-based prospective memory lapses are now well established in AD, with disruptions evident across the various stages required for prospective memory success (for a meta-analytical review, see [208]). Such lapses bear obvious relevance to instrumental activities of daily living in dementia and represent an important area of research given their widespread functional implications, for instance in medication adherence or keeping medical appointments [209,210]. AD patients display an impaired capacity to execute intentions during prospective memory tasks, such as an instruction to tell an administering clinician that it is time for a break after 15 minutes have passed. This deficit is compounded by an inability to remember the appropriate action, particularly when this must be spontaneously retrieved [202,211]. Task-based prospective memory difficulties are corroborated by carer reports, whereby AD patients display a range of prospective memory disturbances in their daily lives, such as deciding to do something in a few minutes’ time and then forgetting to do it [212]. These difficulties have been found to relate to cognitive mechanisms such as episodic memory integrity and grey matter intensity decrease in prefrontal, medial temporal, and posterior parietal regions [212,213]. Many of the regions implicated in time-based prospective memory overlap with those known to support episodic past and future mental time travel [202], raising the possibility that these functions might decline in parallel in AD (see [132]). A number of recent studies have also begun to illustrate a specific role for episodic simulation in selectively boosting prospective memory performance in both health and disease [214,215,216,217,218,219,220,221], while related research demonstrates that elaborative encoding via implementation intention framing holds promise for enhancing prospective memory performance in dementia [222].

Time-based prospective memory is, unsurprisingly, also compromised in bvFTD, resonating with a large body of literature pointing to the importance of the frontal lobes for prospective memory performance across various stages, including maintaining and retrieving intentions at the right moment (e.g., [223]). Only a handful of studies have formally investigated time-based prospective memory in bvFTD, and these have demonstrated performance deficits of a similar magnitude as observed in disease-matched cases of AD [98]. Interestingly, these impairments were strongly correlated with delayed episodic memory dysfunction in both bvFTD and AD [98,213]. On the neural level, time-based prospective memory impairments in both patient groups reflect prefrontal and medial temporal lobe atrophy [213], reinforcing the central role of the episodic memory system in the origin of prospective memory disturbances in dementia [84].

Prefrontal brain regions appear to be much more heavily implicated in prospective memory disturbances in everyday life in bvFTD. A recent study demonstrated that a primarily prefrontal cortical circuit comprising orbitofrontal, medial prefrontal, dorsolateral prefrontal, and anterior cingulate cortices correlates with carer-rated lapses in prospective memory function in bvFTD [212]. The orbitofrontal cortex emerged as a shared neural correlate across retrospective and prospective memory disturbances in both AD and bvFTD patients, underscoring the importance of this region in the strategic control of memory [212]. Furthermore, under experimental conditions of minimal cognitive demand specifically designed to elicit mind wandering, patients with bvFTD exhibited a tendency towards stimulus-bound cognition, indicative of an increased reliance on external sensory input, akin to what is observed in “environmental dependency syndrome” [82]. This raises the possibility that an inability to decouple from the immediate perceptual surroundings of the patient might compound the difficulties of retrieving intentions when required, resulting in prospective memory failures [1].

The finding of marked impairments in the capacity to carry out intentions at a future point in time in dementia populations is admittedly unsurprising when we consider the vast array of cognitive and neural changes that typify these syndromes, along with the wide range of cognitive capacities called upon in prospective memory. An unexpected and intriguing finding in this context is that of relatively preserved time-based prospective memory in semantic dementia, as has been documented by two independent studies [98,145]. Kamminga et al. administered a shortened version of the Cambridge Behavioural Prospective Memory Test to eight patients with semantic dementia [98]. Participants completed three time-based and three event-based prospective memory tasks while completing simple ongoing filler tasks. Importantly, participants were instructed that they could use whatever tools were available in the room to help them to complete the task, including making written notes of the test instructions using the pen and paper provided, or referring to a large analogue clock that was displayed prominently on the screen of the laptop during testing. The SD patients exhibited preserved time-based prospective memory but impaired event-based prospective memory relative to controls [98]. More recently, La Corte et al. replicated this finding of relatively intact time-based prospective memory in a single case of SD, alongside relatively intact near-future prospection capacity [145]. This raises the question of how patients with marked cortical atrophy and profound cognitive disturbances can successfully perform time-based prospective memory tasks, and whether a compensatory mechanism might be at play (see also [224]).

In time-based prospective memory tasks, a clock is often displayed to facilitate ongoing monitoring processes. Indeed, in the Kamminga et al. study [98], an analogue clock was kept visible throughout the test session, enabling participants to refer to and keep track of time. This availability of external cues to overcome internal difficulties with timing raises a further interesting point regarding the use of compensatory strategies as a form of cognitive offloading. SD patients have been shown to display an increased preoccupation with time, a tendency to “watch the clock”, and an insistence on doing things at a particular time [87]. Clockwatching may in this case prove advantageous to the execution of time-based tasks, but detrimental to event-based tasks, and may, in part, explain the differential profiles of prospective memory performance in SD. However, if the capacity to flexibly use such external cues is disrupted, we would predict an impaired ability to turn to external aids such as clocks to augment task performance.

Recent research on the psychological mechanisms of cognitive offloading has revealed that the flexible use of external memory aids depends on reliable metacognitive insight [14,225,226,227,228]. To strategically adopt a clock-watching strategy requires a degree of awareness about the inaccuracy of one’s internal time perception, and a level of awareness that clock monitoring will be required to successfully fulfil one’s intention. One prediction from this view is that, in dementia syndromes, a relative sparing of insight may be associated with clock monitoring that tracks alongside declines in time perception accuracy. Meanwhile, disruptions to metacognitive insight associated with prefrontal atrophy—as in bvFTD—might break the link between time perception difficulties and a strategic reliance on external aids. Declines in cognitive flexibility may also play a part, considering that prospective memory tasks require participants to complete ongoing filler tasks. Patients with bvFTD may be less adept at flexibly switching between the competing demands of the filler tasks, clock watching, and implementation of the prospective memory tasks, resulting in difficulties in strategically relying on external aids when most needed. Collectively, these avenues represent important areas for future investigation to arrive at a comprehensive understanding of the various mechanisms driving prospective memory impairments in dementia.

## 7. Improving Measurement and Assessment

Given the functional significance of subjective timing disturbances in everyday life, it is imperative that the field move towards objective measurement and improved clinical assessment of these constructs [229]. Despite a proliferation of time-perception tasks in the literature, it remains unclear how task design influences performance, or how subjective timing complaints in dementia can be quantified in an objective manner. Reconciling the precise relationship between behavioural performance in time-perception tasks and patient- or carer-reported clinical symptoms—such as disorientation—further represents an important avenue for research. A similar argument can be made in relation to the assessment of mental time travel, as existing techniques are inherently difficult to validate [230]. An important goal will be to develop measures of mental time travel that are both psychometrically sound and clinically useful, potentially enhancing the capacity to screen for early cognitive changes in dementia (e.g., [231]). One promising avenue is the use of refined coding protocols for narrative scoring that index the intersection of episodic and semantic elements during mental time travel [69,232]. Finally, for both time perception and mental time travel, we note that existing measurement and assessment tools are overwhelmingly laboratory-based, limiting their ecological and clinical validity. The development of ecologically valid tasks to tap the real-world relevance of subjective timing impairments in dementia represents the critical next step to quantify such disturbances and, ultimately, support the individual in their everyday activities of living.

## 8. Conclusions

Subjective timing is a multifaceted and inherently complex cognitive capacity, vulnerable to disruption across dementia syndromes. This vulnerability is evident not only in terms of the perception of short intervals, but also the capacity to mentally traverse longer stretches of time across past and future temporal contexts. Here, we have explored how neurodegeneration influences the cognitive profile of time perception and mental time travel, using AD and FTD as representative conditions to explicate the mechanisms and consequences of impairment. We hope that this narrative review will serve simultaneously as an overview of basic science questions around neurodegeneration-related changes to these vital human timing capacities, as well as a tool for scientists and clinicians seeking insight into the subjective time disruptions displayed by their patients. While we have seen that subjective time disturbances are common in dementia, further research is needed before the potential transdiagnostic implications of these disturbances can be thrown into sharp relief. To illustrate the functional consequences of disruptions to subjective timing, we focused here on two illustrative adaptive capacities: intertemporal decision-making and prospective memory. In both cases, declines to either time perception or mental time travel have profound consequences for future-directed behaviours. Future work charting the interplay between time perception and mental time travel in these domains stands to greatly deepen our knowledge of the cognitive mechanisms underlying prospective functions in everyday life. At the same time, targeted efforts to uncover such neurocognitive mechanisms may also prove fruitful in helping families, caregivers, and individuals with dementia to navigate disruptions to their fundamental capacities for subjective timing.
